# *S* haplotype collection in Brassicaceae crops—an updated list of *S* haplotypes

**DOI:** 10.1270/jsbbs.22091

**Published:** 2023-05-17

**Authors:** Masaya Yamamoto, Tomoko Ishii, Marina Ogura, Takashi Akanuma, Xing-Yu Zhu, Hiroyasu Kitashiba

**Affiliations:** 1 Graduate School of Agricultural Science, Tohoku University, 468-1 Aramaki Aza Aoba Aobaku, Sendai, Miyagi 980-8572, Japan

**Keywords:** self-incompatibility, *S* haplotype, *S*-locus receptor kinase, *S*-locus cysteine-rich protein/*S*-locus protein, *S*-locus glycoprotein, *Brassica*, *Raphanus*

## Abstract

Self-incompatibility is the system that inhibits pollen germination and pollen tube growth by self-pollen. This trait is important for the breeding of *Brassica* and *Raphanus* species. In these species, self-incompatibility is governed by the *S* locus, which contains three linked genes (a set called the *S* haplotype), i.e., *S*-locus receptor kinase, *S*-locus cysteine-rich protein/*S*-locus protein 11, and *S*-locus glycoprotein. A large number of *S* haplotypes have been identified in *Brassica oleracea*, *B. rapa*, and *Raphanus sativus* to date, and the nucleotide sequences of their many alleles have also been registered. In this state, it is important to avoid confusion between *S* haplotypes, i.e., an identical *S* haplotype with different names and a different *S* haplotype with an identical *S* haplotype number. To mitigate this issue, we herein constructed a list of *S* haplotypes that are easily accessible to the latest nucleotide sequences of *S*-haplotype genes, together with revisions to and an update of *S* haplotype information. Furthermore, the histories of the *S*-haplotype collection in the three species are reviewed, the importance of the collection of *S* haplotypes as a genetic resource is discussed, and the management of information on *S* haplotypes is proposed.

## Introduction

**Fig. 1.**
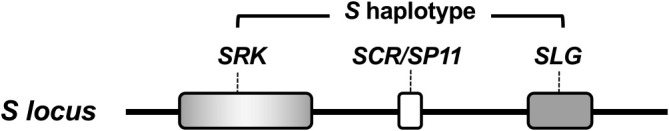
Linkage of *SRK*, *SCR/SP11* and *SLG* at the *S* locus. Three genes, *SRK*, *SCR/SP11* and *SLG*, located on the *S* locus and a set of alleles of three genes are called *S* haplotype.

Self-incompatibility is the system that inhibits germination and pollen tube growth by self-pollen. It is present in many flowering plants (de Nettancourt 2001) and was investigated and described by Darwin (1876) in his work. Self-incompatibility has been considered to have evolved to prevent inbreeding depression and maintain genetic variation in populations. Many cruciferous species have a self-incompatibility system. The self-incompatibility has been extensively studied, particularly in *Brassica* and *Arabidopsis* species and their relatives. In this system, incompatible pollen grains are unable to germinate on stigma papilla cells, which are epidermal cells of the stigma, or incompatible pollen tubes cannot penetrate papilla cells. Genetic studies on this trait were performed in the 1950s and revealed that this response is controlled by a single locus, the *S* locus, with multiple alleles (Bateman 1955). The self-incompatibility phenotype of pollen and the stigma is sporophytically determined by the diploid *S*-genotype of the parent plant. Genetic dominance relationships between *S* alleles have been observed in both the stigma and pollen (Hatakeyama *et al.* 1998a, Thompson and Taylor 1966). Biochemical and molecular genetic studies revealed three important genes at the *S*-locus, two of which are key factors for self-recognition in the self-incompatibility response: *S*-receptor kinase (*SRK*) as a female determinant (Stein *et al.* 1991) and *S-locus cysteine rich protein/S-locus protein 11* (*SCR/SP11*) as a male determinant (Schopfer *et al.* 1999, Suzuki *et al.* 1999). The third gene is *S*-locus glycoprotein (*SLG*), which is highly similar to the extracellular domain (*S*-domain) of SRK (Nasrallah *et al.* 1987). These three genes are tightly linked to each other at the *S*-locus and their alleles are inherited by progeny as one set. A genetic set of the alleles of these genes at the same *S* locus is designated as ‘*S* haplotype’ (Nasrallah and Nasrallah 1993) (Fig. 1). The term ‘*S* haplotype’ is used in this review, because ‘*S*-allele’ and ‘*S*-variant’ used in previous studies corresponds to ‘*S* haplotype’.

A large number of *S* haplotypes have been collected in major *Brassica* and *Raphanus* vegetable species, i.e., *Brassica oleracea*, *B. rapa*, and *Raphanus sativus*, with nucleotide sequence information on *SRK*, *SCR/SP11*, and/or *SLG* alleles (Cho and Kim 2022, Oikawa *et al.* 2011). Based on the extent of sequence similarity shared by the *SLG* and *SRK* alleles, *S* haplotypes are grouped into two major classes, class I and II (Nasrallah *et al.* 1991). Genome resequencing has recently progressed in many inbred lines and cultivars of these species. These projects allow us to discover new *S* haplotypes. Furthermore, novel *S* haplotypes are expected to be found in uninvestigated resources. Actually, we newly identified four *S* haplotypes in *B. rapa*. Therefore, the number of identified *S* haplotypes is expected to further increase. Under this forecasted case, it is important to avoid confusion between *S* haplotypes, i.e., an identical *S* haplotype with different names and a different *S* haplotype with an identical *S* haplotype number. Therefore, we revised and updated *S* haplotype information on these three species and constructed a list on the Tohoku University Brassica Seed Bank website, which is easily accessible for the nucleotide sequence information of the latest *S*-haplotype genes. In this paper, firstly we review the historical processes of the collection and identification of *S*-haplotypes in these species. Next, we add information on eight novel *S* haplotypes that we newly identified. The nucleotide sequences of the newly identified alleles of known *S* haplotypes and information on novel *S* haplotypes collected from genome resequences have also been added to the list. Moreover, the development of DNA markers to identify or discriminate between *S* haplotypes is reviewed. Genome resequences provide information on the *SP11*-*methylation-inducing* region (*SMI*) and *SMI2*, which are factors that induce the epigenetic control of dominance relationships on the pollen side. This information will facilitate investigations on and predictions of dominance relationships between *S* haplotypes. Therefore, we herein describe discovered *SMI* and *SMI2* sequences. In the end, we discuss the importance of the collection of *S* haplotypes as a genetic resource and propose the management of information on *S* haplotypes and *S*-tester lines.

The alleles of *S*-locus genes and *S* haplotypes have been commonly represented by numbers. According to standard nomenclature, alleles are shown with “+” or “–”, e.g., *S*+*1* or *S-1* (Meinke and Koornneef 1997, Østergaard and King 2008). To designate alleles of the *S*-locus and *S* haplotypes, “–” followed by Arabic numerals is hereafter used. Additionally, letters that abbreviate the species name are prefixed to the *S* haplotypes and alleles: e.g., *S* haplotypes of *B. rapa* are represented by *BrS-1*.

## Collection and unification of *S* haplotypes

At the beginning of the collection of *S* haplotypes, the *S* genotype of a plant was identified based on a pollination assay. Selfed seeds were obtained through bud pollination from a plant, and a family was then produced. The *S* genotype of each plant in a family was identified by self- and cross-compatibility in intra-family diallel pollinations. Compatibility was determined based on the seed set or pollen tube behavior on the stigma through observations by fluorescence microscopy. Following this step, the *S* homozygotes produced by each family were subjected to diallel pollinations in order to identify the *S* genotype, and the *S* homozygote with the independent *S* haplotype was then a *S*-tester line. After the establishment of *S*-tester lines, the *S* genotyping of unidentified plants was conducted by test crossing with *S*-tester lines. This became the standard method for identifying *S* haplotypes.

To reduce the time and labor associated with the handling of many *S*-tester lines, an isoelectric focusing analysis of *S* glycoproteins (=SLG) was developed to identify *S*-haplotypes (Nishio and Hinata 1978, 1980, Nou *et al.* 1993a, 1993b). After the cloning of *SLG* genes, a genomic Southern blot analysis of *SLG* was used to identify *S*-haplotypes (Nasrallah *et al.* 1988, Okazaki *et al.* 1999). Furthermore, a PCR-RFLP analysis of *SLG* was developed (Brace *et al.* 1993, Nishio *et al.* 1994). This method has been useful for discovering new *S* haplotypes and discriminating between *S* haplotypes. Many alleles of *SCR*, *SRK*, and *SLG* have been isolated due to the development of many primer sets, and their nucleotide sequences have been elucidated. Through the methods described above, the collection of *S* haplotypes in the following species has progressed: *B. oleracea*, *B. rapa*, and *R. sativus*.

### *B. oleracea* 

The collection of *S* alleles (‘*S* haplotype’ is currently the proper term) was started for *B. oleracea* in the 1960s by Dr. Thompson’s group (Plant Breeding Institute, Cambridge, England) and Dr. van Hal’s group (Unilever Research, Holland). The *S*-tester lines of 23 *S* haplotypes from marrow stem kale and 15 *S* haplotypes from Brussels sprout were established by the former group (Thompson 1968) and the latter group (van Hal and Verhoven 1968), respectively. By 1973, 47 *S*-tester lines were produced by Dr. Thomson and transferred to Dr. Ockendon’s group, i.e., the National Vegetable Research Station at Wellsbourne. Since the 47 *S*-tester lines included redundantly identical *S* haplotypes, Dr. Ockendon spent several years solving this issue and successfully discriminated between 36 independent *S* haplotypes using pollination tests and the production of *S-*tester lines for these *S* haplotypes. In this collection, the *S*-haplotype number designated by Dr. Thompson was inherited. The four *S* haplotypes discovered by van Hal and not found in Thompson’s collection were referred to as *S-60* to *S-63* (Ockendon 1974). Information on 40 *S* haplotypes is listed in the study by Ockendon (2000). From 1975 onwards, Ockendon’s group continued to search *S* haplotypes in *B. oleracea* crops, such as broccoli, cabbage, and wild forms (Ockendon 1980, 1982, Stern *et al.* 1982), and newly identified and added 10 *S* haplotypes to the list, resulting in 50 *S* haplotypes (Ockendon 2000). These *S*-tester lines were transferred to Dr. Nishio’s group in Tohoku University, Japan.

Using *S*-tester lines and F_1_ cultivars, Dr. Nishio’s group cloned and elucidated the full or partial sequences of the *SLG*, *SRK*, and *SCR/SP11* alleles of many *S* haplotypes (summarized in Oikawa *et al.* 2011) as shown in Table 1, i.e., 41 *S* haplotypes; however, unidentified alleles remained. Together with information on several *S* haplotypes identified by other groups, the *SLG*, *SRK*, or *SCR/SP11* alleles were identified in 45 *S* haplotypes by 2011 (Oikawa *et al.* 2011, Sato *et al.* 2002). In parallel with their identification, the re-production of *S*-tester lines advanced to solve late flowering in specific *S*-homozygous lines of Ockendon (2000), i.e., *S-3*, *-8*, *-9*, *-12*, *-22*, *-23*, *-29*, *-46*, *-58*, *-60*, and *-62* (Oikawa *et al.* 2011). These lines were crossed with the broccoli cultivar ‘Ryokurei’ and respective *S*-homozygous lines were selected from their offspring. A series of *B. oleracea* *S*-tester lines (Table 1) are stored in a laboratory of Plant Breeding and Genetics at Tohoku University in Japan.

### *B. rapa* 

In the 1970s, Dr. Hinata and colleagues in Tohoku University produced *S*-homozygous lines from a wild population collected at the Oguni-machi area of Yamagata prefecture in Japan. Thirteen *S*-homozygous lines, *S-1* to *S-13*, were developed (Kitashiba and Nishio 2013, Nishio and Hinata 1978). These lines were subjected to an analysis of *S* glycoproteins in the stigma by isoelectric focusing (Nishio and Hinata 1978). After this analysis, three lines, i.e., *S-8*, *S-9*, and *S-12*, were frequently used and played a significant role in the elucidation of the molecular mechanisms underlying self-incompatibility genetically, physiologically, and biochemically (Schopfer *et al.* 1999, Suzuki *et al.* 1999, Takayama *et al.* 2001). After the collection of *S* haplotypes, Dr. Hinata’s group newly identified 15 *S* haplotypes from an independent wild population collected at another spot of Oguni-machi and gave a number to each *S* haplotype, i.e., *S-22*, *S-25*, *S-26*, *S-28*, *S-29*, *S-34*, *S-35*, *S-36*, *S-38*, *S-39*, *S-41*, *S-44*, *S-46*, *S-45*, and *S-99* (Nou *et al.* 1993a, 1993b). *S-28*, *S-34*, and *S-26* were speculated to be identical to *S-9*, *S-5*, and *S-7*, respectively, based on a diallel pollination assay and immunoblot analysis (Nou *et al.* 1993b). At this time, the renewed *S* numbers *S-43*, *S-24*, and *S-42* were given to *S-8*, *S-12*, and *S-13*, respectively. Dr. Hinata’s group also surveyed another population collected at the Balcesme area in Turkey and 16 *S* haplotypes were identified by a pollination assay and *S*-locus glycoprotein analysis (Nou *et al.* 1993a). *S*-homozygous lines were subjected to a diallel pollination assay together with the 18 *S*-homozygous lines developed from Oguni-machi, and 29 *S* haplotypes were ultimately estimated in the examined materials (Nou *et al.* 1993b). In this experiment, eleven *S* haplotypes, i.e., *S-21*, *S-23*, *S-30*, *S-31*, *S-32*, *S-33*, *S-37*, *S-40*, *S-47*, *S-48*, and *S-49*, which were identified from Balcesme, were newly numbered. In a series of experiments by Nou *et al.* (1991, 1993a, 1993b), four *S* haplotypes, i.e., *S-22*, *S-24*, *S-35*, and *S-42* (*S-13*) were common between the Japanese and Turkey populations.

In the 1990s, the identification of the sequences in most *SLG* alleles and several *SRK* alleles was advanced by Japanese researchers (Hatakeyama *et al.* 1998b, Kusaba *et al.* 1997, Watanabe *et al.* 1994, Yamakawa *et al.* 1994, summarized in Oikawa *et al.* 2011). In 1999, two new *S* haplotypes, *S-52* and *S-60*, were added by a pollination assay and the identification of *SLG* alleles (Takasaki *et al.* 1999). The sequences of the *S* domain and kinase domain of many *SRK* alleles were identified in the 2000s (Fujimoto *et al.* 2006, Okamoto *et al.* 2007, Sato *et al.* 2002, Takuno *et al.* 2007). Regarding *SCR/SP11* alleles, after the identification of the *SP11* allele of the *S-9* haplotype by Suzuki *et al.* (1999) and the *S-8*, *S-12*, and *S-52* haplotypes by Takayama *et al.* (2000), the sequences of the *SCR/SP11* alleles for 15 *S* haplotypes were elucidated (Watanabe *et al.* 2000), followed by the sequencing of many *SCR/SP11* alleles in *B. oleracea* (Kimura *et al.* 2002, Sato *et al.* 2002). In the latter half of the 2000s, Dr. Nishio’s group newly identified 16 *S* haplotypes from resources stored in the Tohoku Univ. *Brassica* Seed Bank, Kaneko Seed Company (Japan), and IPK Gene Bank (Gatersleben, Germany) mainly by the sequencing of *SCR/SP11* alleles (Fujimoto *et al.* 2006, Oikawa *et al.* 2011, Takuno *et al.* 2007, 2010) and assigned *S-53* to *S-56* and *S-61* to *S-72*. The *SLG* sequences of old dead seeds of the original *S*-tester lines (Nishio and Hinata 1978) were revealed to correspond to reported nucleotide sequences (Kitashiba and Nishio 2013). In addition, *S-31* and *S-39* were the same as *S-29* and *S-40*, respectively. On the other hand, the *S-23* and *S-42* lines had been lost and any sequences of *SLG*, *SRK* and *SCR/SP11* alleles were not elucidated. Either *SLG*, *SRK*, and *SCR/SP11* alleles have been identified in total of 48 *S* haplotypes in *B. rapa* (Table 2).

While the alleles of many *S*-haplotypes as described above were identified, Dr. Nishio’s group re-developed *S*-tester lines for some *S* haplotypes in *B. rapa* to solve the inbreeding depression of *S*-homozygous lines. To achieve this, previously developed *S*-homozygous lines for *S-8*, *S-9*, *S-12*, *S-29*, *S-30*, *S-34*, *S-37*, *S-45*, *S-47*, *S-48*, and *S-49* (Nou *et al.* 1991, 1993a, 1993b) were crossed with an F_1_ cultivar ‘Osome’ and the respective *S*-homozygous lines were selected from their offspring. A series of *B. rapa* *S*-tester lines are stored in a laboratory of Plant Breeding and Genetics at Tohoku University in Japan, although several *S*-tester lines, such as *S-63* and *S-64*, lost the germination ability of seeds.

### *R. sativus* 

*R. sativus* is closely related to *B. rapa* and *B. oleracea*, and also has a self-incompatibility system. Niikura and Matsuura (1997) produced *R. sativus* inbred lines and 17 *S* haplotypes were designated *S-201* to *S-217* through pollination tests. Furthermore, they identified an additional 20 *S* haplotypes by a PCR-RFLP analysis for *SLG* alleles mainly using Japanese landrace cultivars along with East Asian and European radish cultivars, although their *SLG* sequences have not been determined (Niikura and Matsuura 1999).

Dr. Nishio’s group also collected *S* haplotypes in *R. sativus*. They produced inbred lines from F_1_ cultivars and performed a Southern blot analysis using *SLG* probes and a PCR-RFLP analysis (Sakamoto *et al.* 1998). In experiments by Sakamoto *et al.* (1998), thirteen class-I and five class-II *S* haplotypes were identified and *S-1* to *S-18* were assigned to the inbred lines used. In addition, the *SLG* alleles of *S-1* to *S-8* were sequenced (Sakamoto *et al.* 1998). Using the same materials along with two *S*-homozygous lines for *S-19* and *S-21*, Okamoto *et al.* (2004) determined the nucleotide sequences of the second exon of *SCR/SP11* encoding the mature protein in ten *S* haplotypes, i.e., *S-1*, *S-2*, *S-4*, *S-6*, *S-8*, *S-9*, *S-14*, *S-18*, *S-19*, and *S-21* as well as those of the *S*-domain of *SRK* alleles in nine *S* haplotypes (*S-1*, *S-2*, *S-4*, *S-6*, *S-8*, *S-9*, *S-18*, *S-19*, and *S-21*). The class-I *S* haplotypes, *S-1* to *S-8*, *S-14*, *S-18*, *S-19*, and *S-21* along with the class-II *S* haplotypes, *S-9*, *S-11*, and *S-15* were identified with the sequences of either gene of the *S* haplotypes. In 2018, further nucleotide sequencing was performed using the same materials along with newly developed *S*-homozygous lines (Haseyama *et al.* 2018). This additional analysis resulted in the identification of 24 *S* haplotypes by 2018, i.e., *S-1* to *S-9*, *S-11*, *S-14*, *S-15*, *S-17* to *S-19*, *S-21* to *S-23*, *S-25*, *S-26*, and *S-28* to *S-31*. In which, 23 *SCR/SP11* alleles, 19 *S*-domains, and 14 kinase domains of 19 *SRK* alleles and 18 *SLG* alleles were identified. On the other hand, Lim *et al.* (2002) reported the nucleotide sequences of nine *SLG* alleles and independently assigned them as *S-1* to *S-10*. However, the *SLG* sequences of *S-6*, *S-2*, and *S-7* by Lim *et al.* (2002) were highly similar to those of *S-1*, *S-2*, and *S-4*, respectively, by Sakamoto *et al.* (1998), with 100% or more than 99% similarity at the deduced amino acid sequences (Nishio and Sakamoto 2017). Furthermore, Lim’s group at the National Horticultural Research Institute (Korea) deposited eight *SLG* sequences in a public DNA database in 2004. *S-12* and *S-18* assigned by the group were found to be identical to *S-3* and *S-6*, respectively, by Sakamoto *et al.* (1998) (Nishio and Sakamoto 2017). Five *SCR*-allele sequences were registered in the database with the *S*-haplotype names of *S-1* to *S-5* by Kim’s group at Sunchon National University (Korea) in 2003. Similarly, the *SCR* sequences of both *S-4* and *S-5* were highly similar to that of *S-9* by Okamoto *et al.* (2004), while the *SCR* sequences of *S-1*, *S-2*, and *S-3* were almost identical to those of *S-26*, *S-15*, and *S-29* by Haseyama *et al.* (2018). As explained above, the same or highly similar sequences were reported with different *S* haplotype names, and different sequences were also registered with the same *S*-haplotype names. In contrast to cases in *B. rapa* and *B. oleracea*, the confusion of *S*-haplotype names occurred in *R. sativus*. To overcome this issue, the unification of *S*-haplotype names was performed by comparisons of the sequences between alleles (Haseyama *et al.* 2018) because Nishio’s group identified most of the *SCR/SP11*, *SRK*, and *SLG* sequences from the majority of the *S* haplotypes they collected. Following this unification, Wang *et al.* (2019b) identified 15 *S* haplotypes in Chinese local varieties, including three new *S* haplotypes that were named based on the list by Haseyama *et al.* (2018). In 2022, Cho and Kim (2022) integrated the 27 *S* haplotypes, which were previously identified in the Korean breeding lines by *SLG* and *SRK* nucleotide sequencing (Kim and Kim 2019), into the unified list. In 2022, 40 *S* haplotypes have so far been identified (Table 3).

## Updated and comprehensive lists of *S* haplotypes in *B. oleracea*, *B. rapa*, and *R. sativus*

### Survey of *SRK*, *SCR*, and *SLG* genes in public databases

To update previous lists of the *B. oleracea*, *B. rapa*, and *R. sativus* *S* haplotypes as described above, the available nucleotide sequences of *SRK*, *SLG*, and *SCR* of these three species were searched in published studies and public databases. In the designation of *S* haplotypes and the alleles of *S* haplotypes in each species, letters that abbreviate the species name are hereafter prefixed to the *S* haplotypes and alleles: e.g., the *S-1* haplotype of *B. rapa* is represented by *BrS-1* and the *SLG* allele of *BrS-1* by *BrSLG-1*. Comparisons among *SCR* sequences revealed that the *S-x* and *S-z* haplotypes of *B. rapa* (Isokawa *et al.* 2010) were identical to *BrS-61* and *BrS-70* of *B. rapa* (Takuno *et al.* 2010), respectively (Table 2). In radish, eight *S* haplotypes in *R. raphanistrum* (*Rr*), which is a close relative to *R. sativus*, were deposited in the public database (Supplemental Table 1). Two of the *R. raphanistrum* *S* haplotypes, namely, *RrSCR-6* and *RrSCR-8*, were highly similar to *RsSCR-15* (99%) and *RsSCR-26* (95%), respectively.

### Identification of novel *B. rapa* and *R. sativus* *S* haplotypes

We recently identified novel *S* haplotypes from *Brassica* and *Raphanus* species. Four nucleotide sequences of *SLG* were identified from the following materials: *B. rapa* Fast Plants (In The Woods, Aomori, Japan), *B. rapa* variety *utilis* (Watanabe Seed Co., Miyagi, Japan), and the *B. rapa* C-121 accession (Tohoku University Brassica Seed Bank) (see Supplemental Text 1), and were designated as *BrSLG-75*, *BrSLG-76*, *BrSLG-77*, and *BrSLG-78* (Table 2). A *BrSCR-77* sequence was also identified (Table 2). In *R. sativus*, three novel nucleotide sequences of *SLG* were identified and designated as *RsSLG-53*, *RsSLG-54*, and *RsSLG-55* following the last number given by Cho and Kim (2022). Moreover, a novel *SCR* sequence (*RsSCR-56*) was identified from a *R. sativus* cultivar stored in the National Agriculture and Food Research Organization Genebank Project (Genebank JP Number 26992 and 26993) (Table 3). Overall, four novel *B. rapa* *S* haplotypes and four novel *R. sativus* *S* haplotypes were discovered.

New *SLG* and *SCR* nucleotide sequences of known *S* haplotypes were identified. In the *BrS-62*, *BrS-63*, *BrS-66*, *BrS-67*, *BrS-70*, *BrS-71*, and *BrS-72* haplotypes, only the *SCR/SP11* alleles of the *S*-haplotype genes were determined. Using the same materials as the *S*-tester lines by Nishio’s group, partial fragments of *SLG* alleles containing hyper variable regions (HVR) were amplified and sequenced (see Supplemental Text 1, Table 2). The partial sequence of *BrSLG-71* was highly similar (99.2%) to that of *S locus protein 6* (*SG6*) [accession number JF427568] at the nucleotide and protein levels, respectively, suggesting that *SG6* corresponded to *BrSLG-71*. The *BrSCR-22* gene had a shorter first intron than other class-I *SCR* alleles (Wang *et al.* 2019a). Since *BrS-22* and *BoS-45* are an interspecific pair (Sato *et al.* 2002), the *BoSCR-45* gene was amplified and sequenced using the BrSCR22-F and BrSCR22-R primers, which were designed based on the *BrSCR-22* sequence (see Supplemental Text 1, Table 1). Sequencing revealed that *BoSCR-45* had a short first intron, similar to *BrSCR-22* (MG708357).

Phylogenetic trees of the protein sequences of SCR, the SRK *S* domain and SLG, were constructed (Supplemental Figs. 1–3). A phylogenetic analysis of SCR and the SRK *S* domain showed that seven novel *S* haplotypes (*BrS-75*, *-76*, -*77*, and *-78* and *RsS-53*, -*54*, and -*55*) were class-I *S* haplotypes, while *RsS-56* was a class-II *S* haplotype.

### Identification of *SRK*, *SCR* and *SLG* full-length nucleotide sequences from recent genome information

Next-generation sequencing technology enables the sequencing of many *B. rapa* and *R. sativus* varieties and, thus, provides the full-length nucleotide sequences of *SRK*, *SCR*, and *SLG*. A BLAST (https://blast.ncbi.nlm.nih.gov/) analysis with *BrSCR* as the query sequence revealed that the *B. rapa* *S-60* and *S-22* haplotypes were on chromosome A07 of *B. rapa* subsp. *pekinensis*, the inbred line Chiifu-401-42 genome (Zhang *et al.* 2018), and chromosome A07 of the *B. rapa* FPsc genome (https://phytozome-next.jgi.doe.gov/info/BrapaFPsc_v1_3), respectively. Brara.G02663.1 in the *B. rapa* FPsc genome was similar to the *BrSRK-22* partial sequence, which led to the identification of the full-length nucleotide sequence of *BrSRK-22*. The *B. oleracea* *S-2b* haplotype is on chromosome C06 of the *B. oleracea* var. capitata 02-12 genome (Liu *et al.* 2014).

The radish draft genome contains *RsS-19* haplotypes (Kitashiba *et al.* 2014, Mitsui *et al.* 2015), and the full-length nucleotide sequence of *RsSRK-19* (accession number, KX961694) has been reported by Kim and Kim (2018). Our previous analysis showed that the full-length nucleotide sequences of *RsSCR-19* and *RsSLG-19* were also present in the radish draft genome (Kitashiba *et al.* 2014, Mitsui *et al.* 2015). Cho and Kim (2022) reported that the *S* locus of the *RsS-9* haplotype was located in chromosome 7 (Rs7) of the ‘WK10039’ genome (Jeong *et al.* 2016). We found that the cultivated radish ‘QZ-16’ genome (accession number, PRJEB37015) also contained the *RsS-9* haplotype in chromosome 8 (Chr8), which corresponded to Rs7 reported by Kitashiba *et al.* (2014) and Jeong *et al.* (2016). In the ‘WK10039’ genome, Rs385140.1 and Rs385170.1 corresponded to *RsSLG-9* and the first exon of *RsSRK-9*, respectively (Supplemental Fig. 4A). Two identical *RsSCR-9* genes were found in the ‘WK10039’ genome (Supplemental Fig. 4A). On the other hand, *RsSCR-9* was only located in region 23,318,086–23,317,740 of Chr8 in the ‘QZ-16’ genome. *RsSRK-9* and *RsSLG-9* were located in regions 23,333,706–23,340,153 and 23,369,603–23,370,928, respectively, of Chr8 in the ‘QZ-16’ genome. The coding sequences of *RsSRK-9* and *RsSLG-9* were predicted based on these genome sequences (Table 3). In addition to the *RsS-9* haplotype, *RsSCR-30* in the RUS00293_1 scaffold and *RsSRK-30* and *RsSLG-30* in the RUS00537_1 scaffold were found in the ‘WK10039’ genome (Supplemental Fig. 4B). This result suggested that the *S* genotype of ‘WK10039’ was the *RsS-9/RsS-30* heterozygote. Furthermore, Rs549690.1 and Rs549670.1 corresponded to *RsSLG-30* and the first exon of *RsSRK-30*, respectively (Supplemental Fig. 4B). Two identical *RsSCR-30* genes were located in the RUS00293_1 scaffold (Supplemental Fig. 4B). Based on this analysis, the *RsSRK-30* and *RsSCR-30* coding sequences from the ‘WK10039’ genome were predicted (Table 3).

A BLAST analysis using *RsSCR* sequences revealed that the ‘Okute-Sakurajima’ *R. sativus* cultivar genome (Shirasawa *et al.* 2020) contained the *RsS-6* and *RsS-23* haplotypes. *RSAskr1.0R7g73939* and *RSAskr1.0R7g73939* corresponded to the *S* domain and kinase domain of *RsSRK-23*, respectively. The full-length nucleotide sequence of *RsSCR-23* was found in the region 9,898,692–9,899,212 of Rs7. Consistent with the absence of the amplification of *RsSLG-23* fragments by PCR (Haseyama *et al.* 2018), the *SLG* sequence was not found in Rs7 of the ‘Okute-Sakurajima’ genome. The *S* locus of *RsS-6* was also present in Rs4 of the ‘Okute-Sakurajima’ genome (RSAskr1.0). Although the *RsS-6* locus is assigned at different positions from the *RsS-23* locus, this discrepancy may be attributed to errors in genome assembly because the *S* locus is highly diverse among *S* haplotypes (Fujimoto *et al.* 2006). *RSAskr1.0R4g52319* and *RSAskr1.0R4g52310* are identical to *RsSRK-6* and *RsSLG-6*, respectively. The first exon of *RsSCR-6* was located in region 53,308,717–53,308,777 of Rs4 in RSAskr1.0. The two second exons were found in regions 53,313,905–53,314,083 and 53,324,607–53,324,785 of Rs4 in RSAskr1.0 (Table 3).

### Re-investigation of *RsS-3*, *RsS-4*, *RsS-21*, and *RsS-30* haplotypes

Cho and Kim (2022) reported that the *SRK* kinase domain sequences of the *RsS-21* and *RsS-30* haplotypes (Haseyama *et al.* 2018) showed lower levels of identity (i.e., 94.06 and 85.96%, respectively) than those of the reported *S* haplotypes, i.e., *RsS12* and *RsS1*, by Kim and Kim (2019). However, these inconsistencies were not observed in the *SRK* *S*-domain sequences of the *RsS-21* and *RsS-30* haplotypes. Re-investigations of the nucleotide sequences of the *SRK* kinase domain in the same *RsS-21* and *RsS-30* tester lines as those in Haseyama *et al.* (2018) revealed that the nucleotide sequence of an amplified kinase domain was highly similar (99.9% identity at the nucleotide level) to the *SRK* kinase domain of *RsS1* and mapped to the nucleotide sequence of *RsSRK-30* in the genome of ‘WK10039’ with more than 99.9% identity. A SRK cDNA fragment from *RsS-21* was amplified and nucleotide sequences contained the SRK *S* domain of *RsS-21* (accession number AB114854) and the kinase domain of *RsS12* (Kim and Kim 2019). Based on these findings, we revised sequence information for *RsSRK-21* and *RsSRK-30* as listed in Table 3.

We also noted an error in the sequence of the SRK *S* domain between *RsS-3* and *RsS-4*. We confirmed that the nucleotide sequence of the *SRK* *S* domain of *R. sativus* previously annotated as *RsS-4* (Okamoto *et al.* 2004; accession number AB114848) was actually *RsS-3*. Therefore, the AB114848 designation was revised to correspond to the nucleotide sequence of the *RsSRK-3* *S* domain (Table 3).

We summarized the reported primers for the amplification of the *SCR*, *SRK*, and *SLG* genes in Supplemental Table 2. Either primer combination will amplify the *SCR*, *SRK*, and *SLG* genes or a partial region of these genes.

### Interspecific/intergenic pairs of *S* haplotypes

As the number of identified *S* haplotypes increases, *S* haplotypes with high similarity to those from different species (or genus) in the *SLG*, *SRK*, or *SCR/SP11* sequences have been identified (Kimura *et al.* 2002, Kusaba *et al.* 1997). These pairs are called interspecific/intergenic pairs. Twenty-one interspecific/intergenic pairs have been found by comparisons between *S* haplotypes from *B. rapa*, *B. oleracea*, and *R. sativus* (Haseyama *et al.* 2018, Kimura *et al.* 2002, Kusaba and Nishio 1999, Kusaba *et al.* 1997, Oikawa *et al.* 2011, Okamoto *et al.* 2004, Sato *et al.* 2003, 2004). These pairs are as follows: *BrS-8* (*BrS-43*)/*BoS-32*, *BrS-9* (*BrS-28*)/*RsS-21*, *BrS-12* (*BrS-24*)/*BoS-51*, *BrS-22/BoS-45*, *BrS-25/BoS-14*, *BrS-27/BoS-8/Rs-14*, *BrS-35/BoS-31/RsS-22*, *BrS-32/BoS-68*, *BrS-36/BoS-24*, *BrS-40/BoS-5*, *BrS-41/BoS-64*, *BrS-44/BoS-2b*, *BrS-46/BoS-7*, *BrS-47/BoS-12*, *BrS-49/BoS-61*, *BrS-52/RsS-30*, *BrS-53/BoS-39*, *BrS-54/BoS-28*, *BrS-60/BoS-15*, *BrS-61/BoS-13*, and *BrS-69/RsS-25*. Additionally, according to Supplemental Figs. 1 and 2, high similarity was observed between *SCR/SP11* alleles and between *SRK* alleles in the *BrS-34* and *RsS-17*, *BoS-20* and *RsS-19*, and *BoS-4* and *RsS-23* pairs, suggesting that these three pairs are intergenic pairs. As demonstrated and discussed by Kimura *et al.* (2002), Sato *et al.* (2003, 2004, 2006), and Okamoto *et al.* (2004), each pair is considered to have originated from a common ancestral *S* haplotype. Interspecific/intergenic pairs are useful materials for studies on the domains affecting recognition specificity. Sato *et al.* (2004) found that the sequences of two regions of SCR/SP11, i.e., regions III and V, were highly conserved between interspecific pairs and were predicted to be necessary for the recognition of self SRK. Similarly, these materials will contribute to further studies, such as those on the evolution and diversification of *S* haplotypes.

## Development of *S* haplotype identification using DNA polymorphisms

As the number of identified *S* haplotypes increases, the management of the nucleotide sequences of alleles of *S* haplotypes is important to avoid confusion with *S* haplotype names. Several consensus primer sets have been developed to amplify the fragments of *SLG*, the *S*-domains and kinase domains of *SRK* and *SCR/SP11* from genomic DNA or cDNA (Supplemental Table 2), and nucleotide sequences have been determined as described above. Many *SCR/SP11s* have been amplified by several primers designed from the first exon region as a forward primer and the oligo-dT primer as a reverse primer. The second exon of the *SCR/SP11* sequences were found to be highly diverse among alleles (average 55%; Sato *et al.* 2002). High diversity among alleles was also observed in the regions, i.e., HVR_I, II, and III, of the *S* domains of *SRK* and *SLG*. These findings suggest that the sequencing of *SCR/SP11* and the *S* domains of *SRK* and *SLG* is an effective approach to identify new *S* haplotypes. On the other hand, the kinase domain of *SRK* is the least diverse among alleles. For example, the kinase domains of BrSRK-8 and BrSRK-46 were shown to be almost identical (Kusaba and Nishio 1999). Therefore, if new *S* haplotypes are examined, the cloning and sequencing of the *S*-domains of *SRK*, *SLG*, and *SCR/SP11* are recommended.

Besides sequencing, DNA markers for an analysis of DNA polymorphisms are also very useful for the discrimination or identification of *S* haplotypes. PCR-RFLP markers have been developed based on polymorphisms in *SLG* alleles (Brace *et al.* 1993, Nishio *et al.* 1996) and the kinase domains of *SRK* alleles (Nishio *et al.* 1997, Park *et al.* 2002). Since this method is very simple and reliable, it is generally used for practical breeding, such as F_1_ hybrid breeding programs and seed purity tests, as well as for basic research on self-incompatibility. However, the PCR-RFLP method cannot easily identify the *S* genotypes of heterozygous plants because of complex band patterns. To overcome this issue, Oikawa *et al.* (2011) developed a method to identify 40 and 33 *S* haplotypes in *B. rapa* and *B. oleracea*, respectively. In this method, allele-specific primer sets and allele-specific oligonucleotide probes for each *SCR/SP11* allele were designed from the sequences of each *SCR/SP11* allele. Digoxigenin-labeled DNA fragments were amplified from an unknown *S*-genotype sample using multiplex primer pairs and the products obtained were hybridized with dot-blotted allele-specific probes, resulting in *S*-allele-specific signals. Distinct signals can be detected, even in heterozygous samples. Additionally, the developed allele-specific primer sets (Oikawa *et al.* 2011) are useful for *S*-genotyping. Based on the determined sequences of *SLG* alleles and the *S*-domain of *SRK* alleles, Haseyama *et al.* (2018) recently developed a dot-blot hybridization method for *S*-genotyping using SRK allele-specific probes designed from HVR polymorphisms. Similarly, the magnetic bead hybridization method has been developed to target the HVRs of SRK (Tonosaki *et al.* 2013). These methods will facilitate further research on new *S* haplotypes in an uninvestigated population and the confirmation of the *S* genotyping of materials.

## Dominance relationships between *S* haplotypes

Genetic dominance relationships are observed between *S* haplotypes on the pollen and stigma sides. Thompson and Taylor (1966) and Hatakeyama *et al.* (1998a) reported that class-I *S* haplotypes were generally dominant to class-II *S* haplotypes on the pollen side of *B. oleracea* and *B. rapa*, although dominance relationships were also observed between class-I *S* haplotypes. Shiba *et al.* (2002) reported that transcripts of the *SCR/SP11* allele of the recessive class-II *S* haplotype were not detected in the anther tapetum of *B. rapa* heterozygous plants having class-I and class-II *S* haplotypes. Shiba *et al.* (2006) subsequently showed that the suppression of recessive class-II *SCR/SP11* occurred epigenetically by the *de novo* methylation of the 5ʹ-promoter region. Furthermore, Tarutani *et al.* (2010) found inverted genomic sequences similar to the sequence of the 5ʹ-promoter region in class-II *SCR/SP11* in the flanking region of *SLG* of class-I *S* haplotypes. The inverted repeat sequence, called *SMI*, produced trans-acting small non-coding RNA (small RNA). The transcript *Smi* induced the methylation of the promoters of recessive class-II *SCR/SP11* alleles, resulting in the suppression of class-II *SCR/SP11* transcription.

Four class-II *S* haplotypes (*BrS-44*, *S-60*, *S-40*, and *S-29*) and three class-II *S* haplotypes (*BoS-2b*, *S-5*, and *S-15*) have been reported in *B. rapa* and *B. oleracea*, respectively. These *S* haplotypes show a linear dominance hierarchy on the pollen side in the order of *BrS-44* > *BrS-60* > *BrS-40* > *BrS-29* (Kakizaki *et al.* 2006) and *BoS-2b* > *BoS-5* > *BoS-15* (Thompson and Taylor 1966). Plants heterozygous for combinations of these class-II *S* haplotypes exhibited *de novo* promoter methylation and the suppression of the transcription of recessive *SCR/SP11* alleles in anthers (Shiba *et al.* 2006). Yasuda *et al.* (2016) found another inverted repeat sequence, *SMI2*, on the *S*-locus in *B. rapa*, and demonstrated that the linear dominance hierarchy of *B. rapa* class-II *S* haplotypes was governed by transcripts, i.e., *Smi2*.

The dominance relationships between class-I and class-II *S* haplotypes in *R. sativus* currently remain unknown. Consistent with the findings obtained for *B. rapa*, those on the expression of accession ‘WK10039’ in the Radish Genome Database (http://radish-genome.org) showed that class-I *RsSCR-30* was expressed in anther tissues (Supplemental Fig. 4B), whereas class-II *RsSCR-9* expression was low (Supplemental Fig. 4A). A BLAST analysis against the ‘WK10039’ genome was performed using the stem-loop region of *BrS-9_SMI* as a query sequence. A *BrS-9_SMI*-like sequence (Supplemental Fig. 4C), designated as *RsS-30_SMI* in region 19,931–20,026 of the RUS00537_1 scaffold, was found. This region was located downstream of the 3ʹ untranslated region of *RsSLG-30*, as reported in *B. rapa* *SMI* (Tarutani *et al.* 2010). Three *BrS-9_SMI*-like sequences in the respective two *S*-loci of the ‘Okute-Sakurajima’ genome (Shirasawa *et al.* 2020) and in the RSA1.0_01586.1 scaffold of the ‘Aokubi’ genome (Kitashiba *et al.* 2014) were also found. These sequences were designated as *RsS-6_SMI*, *RsS-23_SMI*, and *RsS-19_SMI*, respectively (Supplemental Fig. 4C). Although a homologous sequence of *BrS-9_SMI* was observed in class-II *S* haplotypes in *B. rapa* (Tarutani *et al.* 2010), *BrS-9_SMI*-like sequences were not found in the class-II *RsS-9* *S* locus in the ‘WK10039’ or ‘QZ-16’ genome. A highly homologous region to the *RsS-30_Smi* sequence was found in the *RsSCR-9* promoter region (Supplemental Fig. 4D), suggesting that recessive class-II *SCR* expression was suppressed by *Smi* produced from the dominant class-I *S* locus in *R. sativus*, similar to observations in *B. rapa* (Tarutani *et al.* 2010).

On the other hand, a *B. rapa* *SMI2*-like sequence was detected in region 23,368,070–23,368,499 of Chr 8 in the ‘QZ-16’ genome. This location corresponds to a region between *SRK* and *SLG*, as observed in *B. rapa* *SMI2* (Yasuda *et al.* 2016). However, the predicted *RsS-9*_*Smi2* precursor had a longer terminal loop than the *B. rapa* *Smi2* precursors (Supplemental Fig. 5). Further expression and functional analyses of *RsS-9*_*Smi2* are needed to establish whether *RsS-9*_*Smi2* suppresses *RsSCR* expression in recessive *S* haplotype(s) in a similar manner to that observed for *B. rapa* (Yasuda *et al.* 2016).

Regarding the dominance relationships of *S* haplotypes in the stigma, genetic analyses of *B. oleracea* and *B. rapa* were performed by Thompson and Taylor (1966) and Hatakeyama *et al.* (1998a), respectively. However, the underlying molecular mechanisms remain unclear. Although Hatakeyama *et al.* (2001) attempted an expression analysis, the recessiveness of *S* haplotypes in the stigma was not associated with lower *SRK* expression levels (Hatakeyama *et al.* 2001), suggesting that dominance relationships are influenced not by differences in the relative expression levels of *SRK*, but by the features of the SRK protein itself. *BrS-8* (*BrS-43*) was co-dominant to *BrS-46* and *BrS-54*, while *BrS-54* was recessive to *BrS-46* on the stigma side. The sequence of a kinase domain of *BrSRK-8* was identical to that of *BrSRK-46* at the amino acid level, and both sequences showed high similarity (98.3%) to that of *BrSRK-54* (Takuno *et al.* 2007). On the other hand, the *S* domains of these SRKs were more divergent (77.8 to 85.3%). These findings suggest the importance of the *S*-domain for the dominance relationships of *SRK*. Furthermore, the possibility that the intensity of affinity between SRKs is involved in dominance relationships was proposed by Naithani *et al.* (2007). However, these inferences are not sufficient to explain dominance relationships. Further genetic and biochemical studies are needed. The collected *SRK* sequences and *S*-tester lines will be helpful for these purposes.

## Future prospects

*Brassica* and *Raphanus* species diversified from a common ancestral species that had been established after whole-genome triplication *ca*. 15 million years ago (Liu *et al.* 2014, Lysak *et al.* 2005, Yang *et al.* 2006). In the ancestral species, a large number of *S* haplotypes were inferred to have evolutionarily arisen and were inherited by each species. The current number of *S* haplotypes is unknown. Nou *et al.* (1993a) estimated the number of *S* haplotypes in *B. rapa* based on the number of *S* haplotypes in wild populations in Japan and Turkey, and more than 100 *S* haplotypes are expected to exist in *B. rapa* species. Wild populations collected in India, Algeria, Egypt, and other places in Turkey are stored in our laboratory at Tohoku University (Tohoku Univ. Brassica Seed Bank), and *S* haplotypes have not yet been surveyed in the resources. Even in a Chinese landrace, the presence of novel *S* haplotypes has been suggested (Takuno *et al.* 2010), although the sequences of these *S* haplotypes remain unknown. Ockendon (2000) predicted that the total number of *S* haplotypes in *B. oleracea* was approximately 50 based on his long-term study of *S*-haplotype collections. By expanding the target for the collection of *S* haplotypes to the same C-genome species as *B. oleracea*, such as *B. incana*, *B. villosa*, and *B. cretica*, novel *S* haplotypes are expected to be discovered. To date, information on *S*-haplotypes in *R. sativus* has mainly been collected from Asian populations and cultivars in Japan, Korea, and China. World *R. sativus* populations are genetically classified into major 4 groups, i.e., the European group, South and Southeast Asian group, East Asian group, including China and Korea, and Japanese group (Kobayashi *et al.* 2020). The *S* haplotypes of radish populations in the European group and South and Southeast Asian group have not yet been surveyed in detail. Therefore, novel *S* haplotypes are expected to be identified in these areas. Although three interspecific pairs in the class-II *S* haplotype between *B. rapa* and *B. oleracea* have been identified, no interspecific pairs have been discovered in the class-II *S* haplotype between *R. sativus* and these species. This suggests that unidentified class-II *S* haplotypes are present in each species. In the exhaustive collection of *S* haplotypes, the development of an efficient method for *S* haplotype identification using the latest techniques and machines, such as a next-generation sequencer, is required. In addition, more recently, information of pangenome sequences published in *B. rapa* and *B. oleracea* species (Belser *et al.* 2018, Golicz *et al.* 2016) has been available for identification of alleles of some genes such as resistance gene analog (RGA) classes (Bayer *et al.* 2019). Although nucleotide sequences of each allele of *S* haplotypes are highly variable, especially *SCR/SP11*, the pangenome information will also contribute to further identification of new *S* haplotypes.

*S* haplotypes have been established through a long evolutionary history. Therefore, identified and unidentified *S* haplotypes are important gene resources and must not be ruined. Although it may be difficult to exchange plant samples, i.e., *S*-tester lines, internationally between countries or institutes due to the Convention on Biological Diversity or the private matters of each research institution, it is necessary to at least manage nucleotide sequence information on *S* haplotypes. Therefore, we opened a website to provide sequence information on *B. rapa*, *B. oleracea*, and *R. sativus* (URL: https://www.agri.tohoku.ac.jp/pbreed/S-haplotype/resource.html). The lists on the website will be useful for comparing a query sequence with the listed *SLG*, *SRK* and *SCR/SP11* sequences. Following the discovery of new *S* haplotypes by researchers, the sharing of their sequences will be very useful. Please let us know the sequences along with deposition in public databases, such as DDBJ, NCBI or GenBank. We will add them to the *S*-haplotype list with references. The sharing of *S*-tester lines is vital. The propagation of our *S*-tester lines of *B. rapa*, *B. oleracea*, and *R. sativus* is in progress and they may be distributed to interested researchers in near future. We also accept deposits of *S*-homozygote or *S*-heterozygote samples of *B. rapa*, *B. oleracea*, and *R. sativus.*

The information collected on *S* haplotypes has contributed to studies on self-incompatibility in Brassicaceae species. However, many questions remain, such as the mechanisms responsible for the intensity and stability of self-incompatibility, dominance relationships between *S* haplotypes on the stigma side and even on the pollen sides, the signal pathway after self-recognition, and the evolution of *S* haplotypes. We expect the further collection and sharing of *S* haplotypes to provide information that will resolve these questions.

## Author Contribution Statement

MY and HK designed the research. All authors preformed the research and analyzed the data. MY and HK wrote the manuscript.

## Supplementary Material

Supplemental Figures

Supplemental Tables

Supplemental Text

## Figures and Tables

**Table 1.** *S* haplotypes of *Brassica oleracea*

*S* haplotype	*SRK*				*SCR*			*SLG*	
	Full	SD	KD		Full	Partial		Full	Partial
*BoS-1*		AB054706*^a^*	AB298889*^b^*			AB476635*^n^*			D85198*^t^*
BoS-2b*	AB024416*^c^*				AB067447*^o^*			AB024415*^c^*	
*BoS-3*	X79432*^d^*				AJ278643*^p^*			X79431*^d^*	
*BoS-4*		AB298890*^b^*	AB298890*^b^*			AB054738*^a^*			
BoS-5*		Y18259*^e^*	Y18259*^e^*		AB067448*^o^*				D88766*^t^*
*BoS-6*	M76647*^f^*				AF195625*^q^*			Y00268*^u^*	
*BoS-7*	AB180898*^g^*				AB180898*^g^*				D85199*^t^*
*BoS-8*		AB054708*^a^*				AB054739*^a^*			AB054727*^a^*
*BoS-9*									D85200*^t^*
*BoS-11*		AB054709*^a^*				AB476636*^n^*			AB326957*^v^*
*BoS-12*	AB180901*^g^*				AB180900*^g^*			AB180902*^g^*	
*BoS-13* (*BoS-13b*)	AB024420*^c^* (AB024422*^c^*)				AF195626*^q^* (EF577028*^r^*)			(AB024418*^c^*)	AB024417*^c^*
*BoS-14*		AB298891*^b^*	AB298891*^b^*			AB054740*^a^*			D85228*^t^*
BoS-15*	AB180903*^g^*					AB089511*^s^*		Y18261*^e^*	
*BoS-16*		AB054710*^a^*	JX416331*^h^*			AJ278640*^p^*			D85202*^t^*
*BoS-17*			AB298892*^b^*			AB476637*^n^*			D85203*^t^*
*BoS-18*	AB032473*^i^*					AB054741*^a^*		^$^AB032471*^i^*	
*BoS-20*		AB054711*^a^*	AB298893*^b^*			AB054742*^a^*			AB054728*^a^*
*BoS-22*									D85229*^t^*
*BoS-23*		AB013720*^j^*	AB013720*^j^*					AB013719*^j^*	
*BoS-24*		AB054712*^a^*	AB298894*^b^*			AB054743*^a^*			
*BoS-25*		AB054713*^a^*	AB298895*^b^*			AB054744*^a^*			D85204*^t^*
*BoS-28*		AB190355*^k^*	AB190355*^k^*		AB190356*^k^*				D85205*^t^*
*BoS-29*	Z30211*^l^*					AB054745*^a^*		X16123*^w^*	
*BoS-31*			AB298896*^b^*			AB476638*^n^*			AB054729*^a^*
*BoS-32*		AB050482*^m^*				AB050480*^m^*			D88765*^t^*
*BoS-33*		AB054714*^a^*	AB298897*^b^*			AB476639*^n^*			AB054730*^a^*
*BoS-35*		AB054715*^a^*	JX416332*^h^*						D85206*^t^*
*BoS-36*		AB054716*^a^*							AB054731*^a^*
*BoS-38*		AB054717*^a^*	AB298898*^b^*						
*BoS-39*		AB054718*^a^*	AB298899*^b^*			AB054746*^a^*			D85207*^t^*
*BoS-45*		AB054719*^a^*	JX416333*^h^*		LC652460				AB054732*^a^*
*BoS-46*						AB054747*^a^*			D85208*^t^*
*BoS-50*		AB054720*^a^*	JX416334*^h^*						AB054733*^a^*
*BoS-51*		AB054721*^a^*	AB298900*^b^*			AB089509*^s^*			D85209*^t^*
*BoS-52*		AB298901*^b^*	AB298901*^b^*			AB476640*^n^*			D85210*^t^*
*BoS-57*		AB054722*^a^*	JX416335*^h^*			AB054748*^a^*			AB054734*^a^*
*BoS-58*		AB054723*^a^*				AB054749*^a^*			AB054735*^a^*
*BoS-60*	AB032474*^i^*							^$^AB032472*^i^*	
*BoS-61*		AB298902*^b^*	AB298902*^b^*			AB476641*^n^*			
*BoS-62*		AB054724*^a^*				AB054750*^a^*			AB054736*^a^*
*BoS-63*			AB298903*^b^*						D85211*^t^*
*BoS-64*		AB054725*^a^*				AB054751*^a^*			D85212*^t^*
*BoS-65*		AB054726*^a^*	AB298904*^b^*						AB054737*^a^*
*BoS-68*		AB298905*^b^*	AB298905*^b^*			AB476642*^n^*			
*BoS-b*		JX840774*^h^*	JX840774*^h^*						
*BoS-c*		JX861859*^h^*							

*^a^* Sato *et al.* 2002, *^b^* Takuno *et al.* 2007, *^c^* Kusaba *et al.* 2000, *^d^* Delorme *et al.* 1995, *^e^* Cabrillac *et al.* 1999, *^f^* Stein *et al.* 1991, *^g^* Fujimoto *et al.* 2006, *^h^* Tian *et al.* 2013, *^i^* Suzuki *et al.* 2000, *^j^* Kusaba and Nishio 1999, *^k^* Fujimoto *et al.* 2004 (unpublished), *^l^* Kumar and Trick 1994, *^m^* Kimura *et al.* 2002, *^n^* Oikawa *et al.* 2011, *^o^* Shiba *et al.* 2002, *^p^* Vanoosthuyse *et al.* 2001, *^q^* Schopfer *et al.* 1999, *^r^* Lan and Li 2007 (unpublished), *^s^* Sato *et al.* 2003, *^t^* Kusaba *et al.* 1997, *^u^* Nasrallah *et al.* 1987, *^v^* Takuno *et al.* 2008, *^w^* Trick and Flavell 1989. * class-II *S* haplotype. *^$^* pseudogene.

**Table 2.** *S* haplotypes of *Brassica rapa*

*S* haplotype	*SRK*				*SCR*			*SLG*	
	Full	SD	KD		Full	Partial		Full	Partial
*BrS-8*	D38563*^a^*				AF195627*^k^*			X55274*^t^*	
*BrS-9*	LC556298	D30049*^u^*	D30049*^u^*		AB022078*^l^*			D30050*^u^*	
*BrS-12*	D38564*^a^*				AB035503*^m^*			D84469*^v^*	
*BrS-21*	AB270775*^b^*					AB039754*^n^*			D85213*^w^*
*BrS-22*	Brara.G02663	AB054061*^c^*	AB054061*^c^*		MG708357*^o^*			AB054060*^e^*	
*BrS-25*		AB298875*^c^*	AB298875*^c^*			AB370002*^p^*			D85214*^w^*
*BrS-26*		AB054691*^d^*				AB039755*^n^*			D85215*^w^*
*BrS-27*		AB054692*^d^*				AB089510*^q^*			D85216*^w^*
BrS-29*	AB008191*^e^*				AB067449*^r^*			AB008190*^e^*	
*BrS-30*		AB054693*^d^*							D85217*^w^*
*BrS-32*		AB054694*^d^*	AB298876*^c^*			AB039756*^n^*			
*BrS-33*		AB054695*^d^*				AB039757*^n^*			N. A.
*BrS-34*		AB054696*^d^*				AB039758*^n^*			D85218*^w^*
*BrS-35*		AB054697*^d^*	AB298877*^c^*			AB370003*^p^*			D85219*^w^*
*BrS-36*		AB054698*^d^*	AB298878*^c^*			AB039759*^n^*			
*BrS-37*		AB054699*^d^*				AB039760*^n^*			D85220*^w^*
*BrS-38*			AB298879*^c^*			AB039761*^n^*			D85221*^w^*
BrS-40*	AB211197*^f^*				AB067450*^r^*				AB054058*^e^*
*BrS-41*		AB054700*^d^*	AB298881*^c^*			AB039762*^n^*			D85222*^w^*
BrS-44*	AB211198*^f^*				AB067451*^r^*				AB054059*^e^*
*BrS-45*	AB012106*^g^*					AB039763*^n^*		AB012105*^g^*	
*BrS-46*	AB013718*^h^*				AB257128*^c^*			AB257128*^c^*	
*BrS-47*	AB180899*^i^*				AB180899*^i^*			AB180899*^i^*	
*BrS-48*		AB054701*^d^*				AB039766*^n^*			D85225*^w^*
*BrS-49*		AB054702*^d^*				AB039767*^n^*			D85226*^w^*
*BrS-52*		AB054703*^d^*	AB298883*^c^*		AB035505*^m^*				AB054815*^x^*
*BrS-53*		AB298884*^c^*	AB298884*^c^*			AB370004*^p^*			AB326958*^y^*
*BrS-54*	AB298592*^c^*				AB298592*^c^*			AB298593*^c^*	
*BrS-55*		AB298885*^c^*	AB298885*^c^*			AB370005*^p^*			AB326959*^y^*
*BrS-56*		AB298886*^c^*	AB298886*^c^*			AB370006*^p^*			AB326960*^y^*
BrS-60*	AB097116*^j^*				AB067446*^r^*			AB097116*^j^*	
*BrS-61*		AB298887*^c^*	AB298887*^c^*			AB543255*^s^*/AB370007*^p^*			AB326961*^y^*
*BrS-62*						AB370008*^p^*			LC652445
*BrS-63*						AB370010*^p^*			LC652446
*BrS-64*						AB370011*^p^*			
*BrS-65*						AB370012*^p^*			
*BrS-66*						AB370013*^p^*			LC652447
*BrS-67*						AB370014*^p^*			LC652448
*BrS-68*						AB370015*^p^*			
*BrS-69*						AB370016*^p^*			
*BrS-70*					AB543254*^s^*	AB370017*^p^*			LC652449
*BrS-71*						AB370018*^p^*		JF427568*^z^*	
*BrS-72*						AB370019*^p^*			LC652450
*BrS-75*									LC652451
*BrS-76*									LC652452
*BrS-77*						LC652455			LC652453
*BrS-78*									LC652454
*BrS-99*		AB054704*^d^*	AB298888*^c^*			AB370009*^p^*			D85227*^w^*

*^a^* Yamakawa *et al.* 1995, *^b^* Okamoto *et al.* 2007, *^c^* Takuno *et al.* 2007, *^d^* Sato *et al.* 2002, *^e^* Hatakeyama *et al.* 1998a, *^f^* Kakizaki *et al.* 2006, *^g^* Hatakeyama *et al.* 1998b, *^h^* Kusaba and Nishio 1999, *^i^* Fujimoto *et al.* 2006, *^j^* Fukai *et al.* 2003, *^k^* Schopfer *et al.* 1999, *^l^* Suzuki *et al.* 1999, *^m^* Takayama *et al.* 2000, *^n^* Watanabe *et al.* 2000, *^o^* Wang *et al.* 2019a, *^p^* Takuno *et al.* 2010, *^q^* Sato *et al.* 2003, *^r^* Shiba *et al.* 2002, *^s^* Isokawa *et al.* 2010, *^t^* Dwyer *et al.* 1991, *^u^* Watanabe *et al.* 1994, *^v^* Yamakawa *et al.* 1994, *^w^* Kusaba *et al.* 1997, *^x^* Takasaki *et al.* 1999, *^y^* Takuno *et al.* 2008, *^z^* Chung *et al.* 2011 (unpublished). * class-II *S* haplotype. N. A. means “not amplified”.

**Table 3.** *S* haplotypes of *Raphanus sativus*

*S* haplotype	*SRK*				*SCR*			*SLG*	
	Full	SD	KD		Full	Partial		Full	Partial
*RsS-1*		AB114846*^a^*	LC341222*^b^*/AY052581*^c^*			AB114836*^a^*		AY052574*^c^*	AB009677*^l^*
*RsS-2*		AB114847*^a^*	LC341223*^b^*/AY052580*^c^*/KX961696*^d^*			AB114837*^a^*		AY052573*^c^*	AB009678*^l^*
*RsS-3*		AB114848*^a^*	KX961702*^d^*			LC325804*^b^*		AY527400*^f^*	AB009679*^l^*
*RsS-4*			LC341224*^b^*/AY052582*^c^*/KX961699*^d^*			AB114838*^a^*		AY052575*^c^*	AB009680*^l^*
*RsS-5*		LC341212*^b^*	LC341225*^b^*			LC325805*^b^*			AB009681*^l^*
*RsS-6*	RSAskr1.0 R4g52319*^e^*	AB114849*^a^*	LC341226*^b^*/AY534536*^f^*		Okute-Sakurajima genome*^e^*	AB114839*^a^*		AY527401*^f^*	AB009682*^l^*
*RsS-7*									AB009684*^l^*
*RsS-8*		AB114850*^a^*				AB114840*^a^*			AB009683*^l^*
RsS-9*	WK10039 genome*^o^*	AB114851*^a^*	KX961695*^d^*		AY422012*^k^*/AY422013*^k^*	AB114841*^a^*		WK10039 genome*^o^*	LC341235*^b^*
RsS-11*		LC341213*^b^*	KX961703*^d^*			LC325806*^b^*			LC341236*^b^*
*RsS-14*						AB114842*^a^*			N. A.
RsS-15*		LC341214*^b^*	LC341214*^b^*/KX961700*^d^*			LC325807*^b^*			N. A.
*RsS-17*		LC341215*^b^*	LC341227*^b^*/KX961698*^d^*			LC325808*^b^*			N. A.
*RsS-18*		AB114852*^a^*	LC341228*^b^*			AB114843*^a^*			LC341237*^b^*
*RsS-19*	KX961694*^g^*	AB114853*^a^*	LC341229*^b^*		Radish draft genome*^p^*	AB114844*^a^*		Radish draft genome*^p^*	LC341238*^b^*
*RsS-21*		AB114854*^a^*	AY534534*^f^*			AB114845*^a^*			N. A.
*RsS-22*		LC341216*^b^*	LC341231*^b^*/AY534535*^f^*			LC325809*^b^*			LC341239*^b^*
*RsS-23*	Okute-Sakurajima genome*^e^*	LC341217*^b^*	LC341232*^b^*/AY534537*^f^*/KX961704*^d^*		Okute-Sakurajima genome*^e^*	LC325810*^b^*		None*^e^*	
*RsS-25*			AY534543*^f^*			LC325811*^b^*		AY534544*^f^*	LC341240*^b^*
RsS-26*	MT241388*^h^*	LC341218*^b^*				LC325812*^b^*			LC341241*^b^*/AY052577*^c^*
RsS-28*						LC325813*^b^*			N. A.
RsS-29*		LC341219*^b^*	KX961708*^d^*			LC325814*^b^*			LC341242*^b^*/AY529652*^f^*
*RsS-30*	WK10039 genome*^o^*	LC341220*^b^*	AY052579*^c^*		WK10039 genome*^o^*	LC325815*^b^*		AY052572*^c^*	LC341243*^b^*
*RsS-31*		LC341221*^b^*	LC341234*^b^*/AY534541*^f^*			LC325816*^b^*			LC341244*^b^*
*RsS-38*		Wang *et al.* 2019b *^i^*	KX961711*^d^*						Wang *et al.* 2019b *^i^*
*RsS-39*		Wang *et al.* 2019b *^i^*	KX961710*^d^*						Wang *et al.* 2019b *^i^*
*RsS-40*			Wang *et al.* 2019b *^i^*						
*RsS-41*			KX961697*^d^*						AY052578*^c^*
*RsS-42*			AY052585*^c^*					AY052576*^c^*	
*RsS-43*	GQ121139*^j^*		AY534533*^f^*/KX961701*^d^*					AY527399*^f^*	
*RsS-44*			AY534538*^f^*/KX961705*^d^*						
*RsS-45*			AY534539*^f^*						
*RsS-46*			KX961706*^d^*						AY529651*^f^*
*RsS-47*			AY534540*^f^*					AY527402*^f^*	
*RsS-48*			AY534542*^f^*						
*RsS-49*			KX961707*^d^*						
*RsS-50*			KX961709*^d^*						
*RsS-51*			KX961712*^d^*						
*RsS-52*			KX961713*^d^*						
*RsS-53*									LC652456
*RsS-54*									LC652457
*RsS-55*									LC652458
RsS-56*						LC652459			
*RsS-201*									Niikura and Matsuura 1997 *^m^*
*RsS-1Zhao*									DQ984139*^n^*

*^a^* Okamoto *et al.* 2004, *^b^* Haseyama *et al.* 2018, *^c^* Lim *et al.* 2002, *^d^* Kim and Kim 2019, *^e^* Shirasawa *et al.* 2020, *^f^* Lim *et al.* 2004 (unpublished), *^g^* Kim *et al.* 2016 (unpublished), *^h^* Kim and Cho 2021 (unpublished), *^i^* Wang *et al.* 2019b, *^j^* Zhou *et al.* 2009 (unpublished), *^k^* Kim *et al.* 2003 (unpublished), *^l^* Sakamoto *et al.* 1998, *^m^* Niikura and Matsuura 1997, *^n^* Zhao *et al.* 2006 (unpublished), *^o^* Jeong *et al.* 2016, *^p^* Kitashiba *et al.* 2014. * class-II *S* haplotype. N. A. means “not amplified”.
