# Supplementary material for: S haplotype collection in Brassicaceae crops—an updated list of S haplotypes
Source: Breed Sci. 2023 May 17;73(2):132–45. doi: 10.1270/jsbbs.22091 (PMC10316313; doi:10.1270/jsbbs.22091)
Supplement: Supplementary file 1 — Supplemental Figures [file 73_132_s1.pdf]

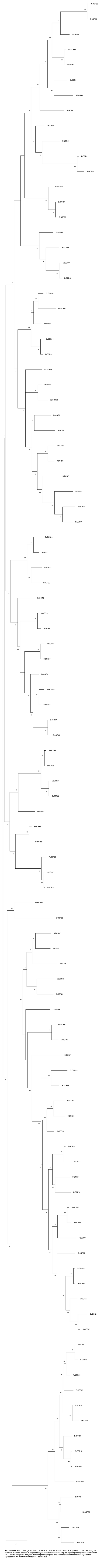

Supplemental Fig. 1. Phylogenetic tree of *B. rapa*, *B. oleracea*, and *R. sativus* SCR proteins constructed using the maximum likelihood method. SCR protein alignment was constructed using the region spanning amino acid residues 19–71 of BrSCR8 (AAF17505) and its corresponding regions. The scale represents the evolutionary distance expressed as the number of substitutions per residue.

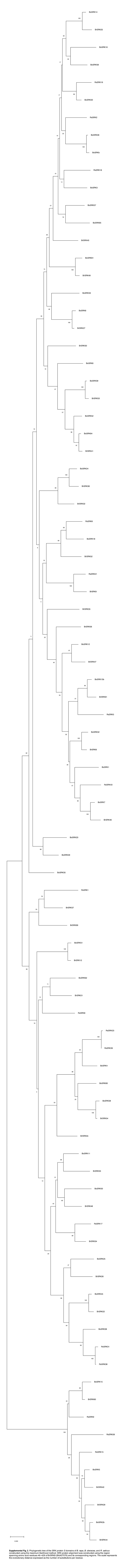

Supplemental Fig. 2. Phylogenetic tree of the SRK protein *S* domains of *B. rapa*, *B. oleracea*, and *R. sativus* constructed using the maximum likelihood method. SRK protein alignment was constructed using the region spanning amino acid residues 46–429 of BrSRK8 (BAA07576) and its corresponding regions. The scale represents the evolutionary distance expressed as the number of substitutions per residue.

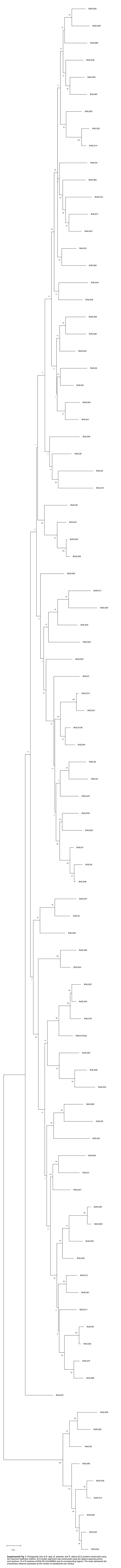

Supplemental Fig. 3. Phylogenetic tree of *B. rapa*, *B. oleracea*, and *R. sativus* SLG proteins constructed using the maximum likelihood method. SLG protein alignment was constructed using the regions spanning amino acid residues 19–419 residues of BrSLG8 (CAA38994) and its corresponding regions. The scale represents the evolutionary distance expressed as the number of substitutions per residue.

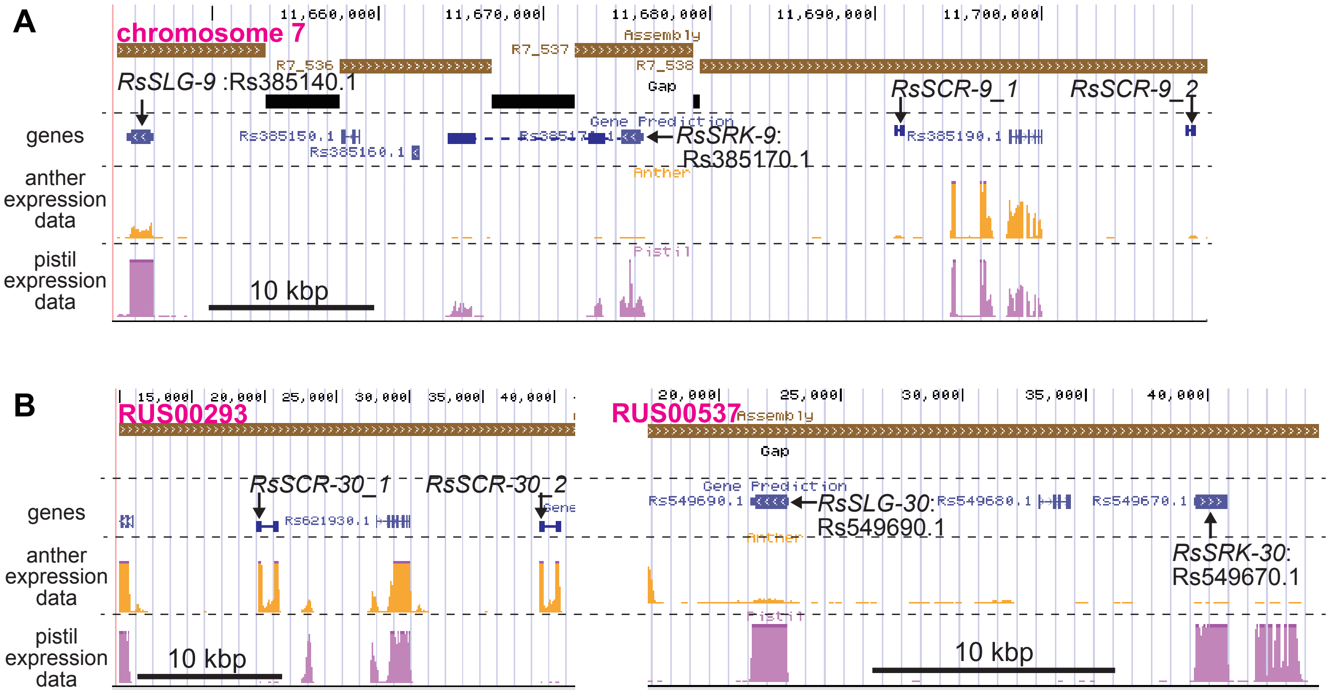

C

```
BrS-9_SMI      ATGCATTAATCGTTTTGTGACTCTTTTACACATGAAGCATAGAATTTGTGGGTTTCTATG      60
RsS-30_SMI     ATGCATTAATCGTTTTGTGACTCTTTTACGCATAAAGCATAAAATTTGTGGGTTTCTATG      60
RsS-6_SMI      ATGCATTAATCGTATTGTGACTCTTTTACGCATAAAGCATAAAATTTGTGGGTTTCTATG      60
RsS-19_SMI     ATGCATTAATCGTTTTGTGACTCTTTTACGCATAAAGCATAAAATTTGTGGGTTTCTATG      60
RsS-23_SMI     ATGCATTAATCGTTTTGTGACTCTTTTATACATAAAGCATAAAATTTGTGGGTTTCTATG      60
               ************* **************   ***  ******* ******************** 

BrS-9_SMI      TTTACGTGTAAAATAGTTACAAAACGATTAGTGCAT      96
RsS-30_SMI     TTTACGTGTAAAATAGTTACAAAACGATTAGTGCAT      96
RsS-6_SMI      TTTACGTGTAAAATAGTTACAAAACGATTAGTGCAT      96
RsS-19_SMI     TTTACGTGTAAAATAGTTACAAAACGATTAGTGCAT      96
RsS-23_SMI     TTTACGTGTAAAATAGTTACAAAACGATTAGTGCAT      96
               ************************************
```

D

```
RsS-30_Smi                5' AUGUUUACGUGUAAAAUAGUUACA 3'
RsSCR-9 5' region   -123 AATATTTACGTGTAAAATAGTTGCAT -100
```

Supplemental Fig. 4. Genome analysis of class-I / class-II *S* locus heterozygotes in the radish accession 'WK10039' . (A and B) *S* locus of *RsS-9* (A) and *RsS-30* (B) haplotypes in the 'WK10039' genome. The genomic region where *RsSRK-9* is located was not sequenced. The expression data of anther and pistil tissues is available at http://radish-genome.org/Genome_browser/. (C) The genomic regions encoding the stem-loop region of *BrS-9_SMI* and its homologous sequences found in *S* locus of class-I haplotype of *R. sativus*. The region encoding the *BrS-9_Smi* and corresponding regions are enclosed within a red rectangle. (D) Sequence alignment of the *RsS-30_Smi* and the *RsSCR-9* 5' region. The text in red indicates mismatched bases compared with *RsS-30_Smi*.

```
RsS-9_SMI2     CTCTTTTGCACGTGAACATTCAGAACACACGTTATTCGTGTATATCTTTGTGACTGATAG   60
BrS-29_SMI2    CTCTTTTGCACGTGAACATTCAGAACACACGTTATTCGTGTATATCTTTGTGACTGATAG   60
BrS-40_SMI2    CTCTTTTTCACGTGAACATTTAGAACACACTTTATTCGTGTATATCTTTGTGACCGATAG   60
BrS-44_SMI2    CTCTTTTTCACGTGAACATTTAGAACACACTTTATTCGTGTATGTCTTCGTGACTGATAG   60
BrS-60_SMI2    CTCTTTTTCACGTGAACATTTAGAACACACCTTATTTGTGTATATCTTTGTGACTGATAG   60
               ******* ************ ********* ***** ****** **** ***** *****

RsS-9_SMI2     ACACACATGTTATATATAAAGAAAACACGCTTGAAAAACTTGTTAAATATAGAATATGT   120
BrS-29_SMI2    ACTCACAAGTAGTCTATAACGTGTTG----------------------------------   86
BrS-40_SMI2    ACTCACATGTAGTCTATAA-----------------------------------------   79
BrS-44_SMI2    ACTCGCATGTAGTCTATAA-----------------------------------------   79
BrS-60_SMI2    ACTCACATGTAGTCTATAA-----------------------------------------   79
               ** * ** **  * *****

RsS-9_SMI2     AATCTATAACGTGTCAGGTAACGACAA-GGTATTCTTGTATAAGGTGTGT-CCTGAATGT   178
BrS-29_SMI2    -----ATAACGTGTCAAGTAACGACAAAGGTATACACGTACAAGGTGTGTTTCTGAATAT   141
BrS-40_SMI2    ---------CGTGTCAAGTAACGACAAAGGTATACATGTATAAGGTGTGTTTTTGAATGT   130
BrS-44_SMI2    ---------CGTGTCAAGTAACGACAAATATATACATGTATAAGGTGTGTTTCTGAATGT   130
BrS-60_SMI2    ---------CGTGTCAAGTAACAACAAATGTATACATGTATAAGGTGTGTTTCTGAATGT   130
                        ******* ***** ****   *** *  *** *********   ***** *

RsS-9_SMI2     TTACGAGTAAAAAG  192
BrS-29_SMI2    TTACGTGTAAAGAA  155
BrS-40_SMI2    TTACGTGTAAAGAA  144
BrS-44_SMI2    TTACGTGTAAAGAA  144
BrS-60_SMI2    TTACGTGTAAAGAA  144
               ***** ***** *
```

Supplemental Fig. 5. Sequence alignment of predicted *RsS-9_SMI2* and *B. rapa SMI2* regions. The mature *Smi2* coding region is enclosed within the magenta rectangle. Arrows indicate the inverted repeat region.
