# Supplementary material for: S haplotype collection in Brassicaceae crops—an updated list of S haplotypes
Source: Breed Sci. 2023 May 17;73(2):132–45. doi: 10.1270/jsbbs.22091 (PMC10316313; doi:10.1270/jsbbs.22091)
Supplement: Supplementary file 2 — Supplemental Tables [file 73_132_s2.pdf]

doi: 10.1270/jsbbs.22091

Supplemental Table 1. *S* haplotypes of *Raphanus raphanistrum*

S haplotype	*SRK*			*SCR*		*SLG*	
	Full	SD	KD	Full	partial	Full	partial
RrS-1		KP117083[a]	KP117076[a]		KP117086[a]		
RrS-2			KP117078[a]				
RrS-3			KP117079[a]		KP117087[a]		
RrS-4		KP117084[a]	KP117081[a]		KP117088[a]		
RrS-5			KP117080[a]				
RrS-6		KP117085[a]	KP117077[a]		KP117089[a]		
RrS-7			KP117082[a]				
RrS-8					KP117090[a]		

[a]Koh 2015 (unpublished).

Supplemental Table 2. List of primers for amplification of *SCR*, *SRK* and *SLG*

Primer name	Direction	Primer sequence (5'→3')
<class-I *SCR* CDS>		
pSP11-1[a]	Forward	ATGAAATCTGCTATTTATGCTTTATTATG
NotI(dT)$_{18}$	Reverse	AACTGGAAGAATTCGCGGCCGCAGGAAT$_{18}$
RT-1[a]	Reverse	ACTGGAAGAATTCGCGGC
pSP11-2[a]	Reverse	TTCATATTCATCGTTTCAAGTC
<class-II *SCR*>		
SPII-F[b]	Forward	CTAGATGTGGGAGCTTGGAAATGCC
40R[c]	Reverse	ATACTGCATAGAGTAACCGTATCTGG
RsSCR-F(II)[d]	Forward	TGACATATGTTCAAGGTAAAYTA
RsSCR-R(II)[d]	Reverse	GATTKAACTTTGCAACAGTAGCAA
<class-I *SLG*>		
PS5[e]	Forward	ATGAAAGGCGTAAGAAAAACCTA
HV-F[b]	Forward	TGGCAAAGTTTCGATTWCCCKAC
PS15[e]	Reverse	CCGTGTTTTATTTTAAGAGAAAGAGCT
<class-II *SLG*>		
PS3[e]	Forward	ATGAAAGGGGTACAGAACAT
PS21[e]	Reverse	GTCAAGTCCCACTGCTGCGGG
< *S* domain of class-I *SRK*>		
PK7[f]	Forward	ATGCAAGGTGTACGATACATCTATCATCATTCTTAC
SRK-S-C1-1 F[g]	Forward	ATGGGTYGCCAACAGAGA
SRK-S-C1-1 R[g]	Reverse	ATTCCGKATATCYGCATTTG
SRK-S-C1-2 R[g]	Reverse	TCCTCCTTATACACCCACCTG
SRK-S-C1-3 F[g]	Forward	TCGTTGGTATCTCGGKATATGG
SRK-S-C1-3 R[g]	Reverse	ACAACCYGTMCCACCATT
SRK-S-C1-1 F[g]	Forward	ATGGGTYGCCAACAGAGA
< transmembrane domain of class-I *SRK*>		
PK1[f]	Forward	CTGCTGATCATGTTCTGCCTCTGG
PK5[f]	Forward	AGACAAAAGCAAGCAAAAGCA
PK8[f]	Reverse	GATCAGAAGAAGCAGAACAGTAACTCCAACAGTC
< kinase domain of class-I *SRK*>		

SRK3-F[h]	Forward	TGATGAGTTATGAAGAGGGA
PK9[f]	Reverse	CCTTGTCCGAGTTTGTTACAGTTGGAGAAATTTTCGG
PK4[f]	Reverse	CAATCCCAAAATCCGAGATCT
PK2[f]	Reverse	ATATTGAATCACTCAGCTATCATA
KD8[i]	Reverse	GCTTTCATATTACCGGGCATCGATGA
SRK-1F[j]	Forward	AGGCTTCAGCATATAAACCTTG
SRK-1R[j]	Reverse	TTACCGGGCATCGATGACTGA
<*S* domain of class-II *SRK*>		
SRK-S-C2 F[i]	Forward	TCGTCTWCAGARTCTCTYACAATC
SRK-S-C2 R[i]	Reverse	CTCTAAWACAGTTACAGTTAGGTGACG
< kinase domain of class-II *SRK*>		
KD4[i]	Forward	GAGGGCGAGAAAGATCTTAATT
KD7[i]	Reverse	AAGACGATCATATTACCGAGC
< *S* domain of class-I *SRK* and *SLG*>		
SLG-1F[j]	Forward	CAACAGAACACTTGTATCTCC
SLG-1R[j]	Reverse	GCCAATCTGACATAAAGATC
< *S* domain of class-I *SRK* and *SLG* >		
SLG-2F[j]	Forward	TCATCCTGCCCTTTCGATCTATGTCAA
SLG-2R[j]	Reverse	TTTCCGCATCTCWACGAGATCTCC

[a]Sato *et al.* 2002, [b]Takuno *et al.* 2010, [c]Sato *et al.* 2006, [d]Haseyama *et al.* 2018, [e]Nishio *et al.* 1996, [f]Nishio *et al.* 1997, [g]Kim and Kim 2019, [h]Delorme *et al.* 1995, [i]Park *et al.* 2002, [j]Wang *et al.* 2019
