# Supplementary material for: S haplotype collection in Brassicaceae crops—an updated list of S haplotypes
Source: Breed Sci. 2023 May 17;73(2):132–45. doi: 10.1270/jsbbs.22091 (PMC10316313; doi:10.1270/jsbbs.22091)
Supplement: Supplementary file 3 — Supplemental Text [file 73_132_s3.pdf]

doi: 10.1270/jsbbs.22091

Supplemental Text 1.

Materials and Methods

Plant materials

Six *S*-tester lines of *B. rapa* (*BrS-62*, *BrS-66*, *BrS-67*, *BrS-70*, *BrS-71*, and *BrS-72*), *B. rapa* Fast Plants (In The Woods, Aomori, Japan), *B. rapa* variety *utilis* (Watanabe Seed Co., Miyagi, Japan), *B. rapa* C-121 accession (Tohoku University Brassica Seed Bank), which were collected from Lagunitas, California, USA, *B. oleracea S*-tester lines of *S-45* (Oikawa *et al*. 2011), and two *R. sativus* cultivars, which were obtained from the National Agriculture and Food Research Organization Genebank Project (Genebank JP Number 26992 and 26993), were used in the present study.

DNA and RNA preparation

Genomic DNA (gDNA) was prepared from leaves using the CTAB method (Doyle and Doyle 1987). Total RNA was prepared from anthers using TRIzol reagent (Thermo Fisher Scientific, Waltham, MA). Isolated total RNA was treated with RQ1 RNase-free DNase (Promega, Fitchburg, WI), and first-strand cDNA was synthesized using the PrimeScript II first strand cDNA Synthesis Kit (TaKaRa Bio, Shiga, Japan), according to the manufacturer's protocol.

Amplification of SRK, SLG, and SCR

BrSLG-62, *-63*, *-66*, *-67*, *-70*, and *-71* PCR fragments were amplified from the gDNA of each *B. rapa S*-tester line with the PS5 and PS15 primers (Nishio *et al*. 1996). *BrSLG-75*, *76*, *78*, and *RsSLG-53* PCR fragments were amplified from the gDNA of *B. rapa* C-121, *B. rapa* Fast Plants, *B. rapa* var. *utilis*, and *R. sativus* (Genebank JP Number 26992),

respectively, with the PS5 and PS15 primers. *BrSLG-72*, *-77*, *RsSLG-54*, and *-55* PCR fragments were amplified from the gDNA of the *B. rapa S-72* tester line, *B. rapa* Fast Plants, *R. sativus* accession (Genebank JP Number 26992), and *R. sativus* accession (Genebank JP Number 26993), respectively, with the HV-F (Takuno *et al*. 2010) and PS15 primers. PCR fragments of the first exon of *BrSRK-22* were obtained from the gDNA of the *B. rapa S-22* tester line with the BrSRK22-F1 and BrSRK22-R2 primers. PCR fragments of the second to seventh exons of *BrSRK-22* were obtained from cDNA prepared from the stigmas of the *B. rapa S-22* tester line with the BrSRK22-F2 and BrSRK22-R1 primers.

BrSCR-77 was amplified using two rounds of reverse transcriptase-PCR from cDNA prepared from the anthers of *B. rapa*. The primer pair of pSP11-1 (Kimura *et al*. 2002) and NotI(dT)$_{18}$ was used for the first round, while the primer pair of pSP11-2 (Kimura *et al*. 2002) and RT-1 long (Okamoto *et al*. 2004) was used for the second round. *BoSCR-45* PCR fragments were amplified from the gDNA of the *B. oleracea S-45* tester line with the BrSCR22-F and BrSCR22-R primers. *RsSCR-56* PCR fragments were amplified from the gDNA of *R. sativus* accession (Genebank JP Number 26992) with the RsSCR-F(II) and RsSCR-R(II) primers (Haseyama *et al*. 2018). The nucleotide sequences of all primers are listed in Supplemental Table 1.

Analysis of nucleotide sequences

PCR products were purified using the NucleoSpin Gel and PCR Clean-up kit (TaKaRa Bio Inc), and sequenced at Eurofins Genomics KK (Tokyo, Japan). Nucleotide sequences were analyzed by Sequencher ver.4.8 (Hitachi Software Engineering, Tokyo, Japan) or CodonCode Aligner ver.9.0.1 (CodonCode Corporation, Centerville, MA, USA).

Phylogenetic trees were constructed using the maximum likelihood method and Jones-Taylor-Thornton model in MEGA version 10.1.8, with 1,000 bootstrap pseudo replicates (Stecher *et al*. 2020).

Literature Cited

Doyle, J.J. and J.L. Doyle (1987) A rapid DNA isolation procedure for small quantities of fresh leaf tissue. Phytochemical Bulletin 19: 11–15.

Stecher, G., K. Tamura and S. Kumar (2020) Molecular evolutionary genetics analysis (MEGA) for macOS. Mol Biol Evol 37: 1237–1239.
